# Provider Perspectives on Group PrEP Care for Sex Workers: A Pre-implementation Study in a U.S. Midwestern Community Health Center

**DOI:** 10.1007/s10508-025-03271-7

**Published:** 2026-01-04

**Authors:** Randi Beth Singer, Jessica Zemlak, Sara Jacobs, Natasha Crooks, Oscar Daniel Bahena, Maya Green, Susan G. Sherman, Geri Donenberg, Alicia K. Matthews, Crystal L. Patil

**Affiliations:** 1 College of Nursing, University of Illinois at Chicago, 845 South Damen Avenue, 812, Chicago, IL 60612, USA; 2 College of Nursing, Marquette University, Milwaukee, WI, USA; 3 Public Health, Northwestern University, Evanston, IL, USA; 4 Onyx Health and Wellness, Chicago, IL, USA; 5 Bloomberg School of Public Health, Johns Hopkins University, Baltimore, MD, USA; 6 Center for Dissemination and Implementation Science, University of Illinois Chicago, Chicago, IL, USA; 7 School of Nursing, Columbia University, New York, NY, USA; 8 School of Nursing, University of Michigan, Ann Arbor, MI, USA

**Keywords:** Sex workers, PrEP care, Centering PrEP, HIV prevention, Group care

## Abstract

Reducing new HIV infections is crucial. Sex workers continue to face disparities in both HIV infection rates and inadequate prevention care. Stigma, criminalization, and financial instability hinder access to vital HIV prevention methods, including Pre-exposure Prophylaxis (PrEP). Evidence-based, community-centered PrEP care may help address these issues. An evidence-based group PrEP care model, Centering PrEP (C-PrEP +), could empower communities and reduce healthcare burdens. This study explored care professionals’ perspectives on barriers and facilitators of C-PrEP + for sex workers. Using the Exploration, Preparation, Implementation, and Sustainment (EPIS) framework, we aimed to develop a pilot implementation plan for C-PrEP + . Individual interviews were held with care professionals at a U.S. Midwestern Community Health Center serving sex workers. To reflect the diverse roles and backgrounds of participants, we interviewed 14 healthcare professionals, including care providers, patient navigators, and billing specialists. Guided by a semi-structured interview guide, we sought care providers’ views on obstacles and enablers related to prescribing PrEP and implementing group PrEP care for sex workers. Using Dedoose, we used directed content analysis to systematically explore their perspectives about C-PrEP + using predefined constructs from the EPIS framework to guide coding and interpretation. Major themes included healthcare for sex workers, challenges in standard PrEP care, perceptions of Centering PrEP, and barriers and facilitators for implementing C-PrEP + . Participants viewed group PrEP care as a means to foster patient-centered approaches and strengthen community ties, while addressing the demands of care. Thoughtful integration of C-PrEP + into clinics may be a promising strategy to improve HIV prevention for sex workers.

## Introduction

Over one million people in the USA live with HIV (Fauci et al., 2019). Declines in incidence are not equitably distributed across populations (HIV & AIDS Trends and U.S. Statistics Overview, 2024) in large part due to social, economic, and demographic factors, including stigma, discrimination, income, education level, and geographical location (Sullivan et al., 2021). Sex workers, individuals who exchange sex for money or nonmonetary items, experience a higher incidence of HIV, driving increased vulnerability and higher incidence of new HIV infections among this population (Benoit & Unsworth, 2022; Carrasco et al., 2017; Getting to Zero Illinois, 2018; Lyons et al., 2020; Maseko et al., 2020; Ramos et al., 2024; Silberzahn et al., 2020; UNAIDS, 2016; World Health Organization, 2014). Guided by the Exploration and Preparation phases of the Exploration, Preparation, Implementation, and Sustainment (EPIS) framework, this study explores provider and staff perspectives of a Centering-informed, community-empowered, group-based PrEP care model (C-PrEP +) designed to meet the PrEP care needs of sex workers (Manant & Dodgson, 2011; Singer et al., 2024). Gaining insight from health staff and practitioners at a Midwestern clinic that serves sex workers aimed to inform a pilot implementation strategy that would be responsive to both sex worker needs and the needs of the health professionals who serve them. The significance of this work lies in its potential to advance culturally safe, community-led, group-based HIV prevention strategies within healthcare systems, with broader implications for health equity and PrEP scale-up for structurally vulnerable populations.

Sex workers face a range of traumatic challenges, encompassing unstable housing, unemployment, incarceration, mental health issues, violence, emotional, physical, and sexual abuse, as well as substance abuse (Froli & Viljoen, 2020, p. 21; Mithani & Judge, 2024; Saulman, 2021). Increased exposure to violence and coercion experienced by sex workers leads to reduced autonomy over healthpromoting behaviors, resulting in frequent engagement in unprotected sex and substance use, heightening their vulnerability to HIV (Baral et al., 2015; Capous-Desyllas & Loy, 2020; Decker et al., 2013; Footer et al., 2020; Lyons et al., 2020; Mithani & Judge, 2024). Despite these hardships, community-empowered interventions, designed, implemented, and evaluated in partnership with sex workers, can improve health service delivery and reduce vulnerability to HIV (Benoit et al., 2017; Bungay et al., 2016; Kerrigan et al., 2015). Community-empowered interventions have proven effective in curbing HIV transmission among sex workers by upholding the foundational ethos of "Nothing about us without us" (Outlaw et al., 2009; Work, 2009). Such interventions prioritize the voices of sex workers throughout all stages of a project, including planning, implementation, evaluation, and dissemination (Benoit et al., 2017; Kerrigan et al., 2015). Historically underserved populations, such as sex workers, face significant barriers to accessing culturally safe healthcare, leading to disparities in sexual health outcomes such as increased rates of HIV and STIs (Benoit et al., 2018; Nerlander et al., 2020). Focusing on clinic settings that are primed for community-driven public health initiatives supports capacity building within the institutions intended to serve those most vulnerable (Clark et al., 2024). Unlike cultural competence, cultural safety does not emphasize developing “competence” through knowledge about the cultures with which professionals are working. Instead, cultural safety emphasizes recognizing the social, historical, political, and economic circumstances that create power differences and inequity in health and healthcare (Curtis et al., 2019). Providers guided by cultural safety understand that cultural competence is insufficient for centering patient needs, especially for those within stigmatized populations. To effectively support activities of daily living and to facilitate harm reduction for sex workers, cultural safety, defined by the patient, and in partnership with the provider, is the goal (Curtis et al., 2019). Providers committed to culturally safe care must also resist commonplace power imbalances by engaging in education and self-reflection to ensure that patient needs remain at the forefront (Curtis et al., 2019; Singer et al., 2022). Our work, adapted and implemented with sex workers as leaders, aligns with this framework (Singer et al., 2021, 2022).

Aligned with Cultural Safety, Centering Healthcare (Centering) is an evidence-based group approach to health promotion that empowers communities, especially those within disadvantaged groups (Rising, 2017; Schellinger et al., 2017; Singer et al., 2024). Centering’s patient-centered method emphasizes social support and collaborative problem-solving, thereby reducing power imbalances found in traditional healthcare. Instead of a one-on-one appointment, a cohort of 8–12 patients meet with the same providers at each visit for regular health assessments, connections to services, followed by 75–90 min of interactive learning and skill-building focused on patients’ experiences (Kershaw et al., 2009; Rising, 2017; Rotundo, 2011). Originally developed for prenatal care, Centering has been adapted for other patient groups and conditions, such as diabetes, sickle cell disease, and wellchild care (Ciribassi & Patil, 2016; Schellinger et al., 2017). The approach has shown positive results, including increased condom use, fewer repeat pregnancies, a lower risk of preterm birth, greater health-related knowledge and satisfaction with care, and more frequent visits (Ciribassi & Patil, 2016; Fuentes-Rivera et al., 2020; Novick et al., 2013; Patil et al., 2013; Schellinger et al., 2017). Centering-informed group care has strong potential for PrEP delivery, as it promotes community empowerment and may be feasible and acceptable for sex workers (Singer et al., 2021, 2024).

Centering’s three well-defined core components, health assessment, interactive learning, and community building, work in tandem to improve health outcomes (Dancy et al., 2014; Patil et al., 2013, 2017; Rotundo, 2011; Schindler Rising et al., 2004). Positive impacts of Centering derive from the group process, which enhances learning, promotes healthy behavior change, builds a sense of control over health, develops a supportive network, and creates a collaborative provider–client relationship through continuity of care (Klima et al., 2009, 2015; Manant & Dodgson, 2011; Novick et al., 2013; Schindler Rising et al., 2004). Additional strengths are Centering’s clear guidelines for replication with fidelity and efficient use of personnel.

Centering PrEP (C-PrEP +) has great potential to integrate into existing health systems and improve the chances of sustainable care. Early community-engaged participatory research provided evidence supporting C-PrEP + and the development of the C-PrEP + facilitator’s guide. In initial work, our team partnered with a clinic, sex workers, community advisory board members, and topical experts to adapt the Centering Healthcare curriculum and related facilitator’s guide for sex workers on PrEP. The clinic, which has 11 locations in Chicago, was already using the approach for prenatal care and was open to adapting it for sex workers starting PrEP. Interventions tested at this clinic have informed practices across clinics nationwide, suggesting considerable potential for scale-up of C-PrEP + .

EPIS is a comprehensive framework that guides the planning and execution of evidence-based practices in real-world settings, recognizing both outer and inner contextual factors (Moullin et al., 2019). The outer context, informed by previous research on sex work and HIV prevention strategies, emphasizes the broader social and policy dynamics and includes societal issues such as criminalization, stigma, and economic inequalities. It highlights structural barriers to effective HIV prevention care for sex workers during the development of interventions. To inform the C-PrEP + model, our foundational work focused on understanding how contextual factors influence the lived experiences of sex workers (Singer et al., 2021, 2022, 2024). Essentially, this foundational work aligns with the exploration and preparation phases of the project.

The inner context, or organizational environment within the healthcare system, includes staff perspectives, organizational culture, and technological challenges. Understanding this context helps identify internal dynamics and barriers within healthcare settings that need to be addressed before implementing C-PrEP + . Combining insights from both the outer and inner contexts will support a comprehensive strategy to implement C-PrEP + and overcome societal and organizational barriers to HIV prevention among sex workers.

## Method

This is a descriptive qualitative study guided by EPIS and Community Empowerment frameworks (Moullin et al., 2019). EPIS provided a structured approach to understand intervention implementation and sustainability over time. Specifically, we applied the Exploration phase to identify contextual barriers and facilitators to group-based PrEP care for sex workers, and the Preparation phase to assess organizational readiness and co-develop an implementation blueprint with key stakeholders. We used EPIS to inform the development of interview guides, coding structure, and thematic analysis, ensuring alignment between stakeholder input and implementation planning (Moullin et al., 2019). Community empowerment emphasized community involvement, peer support, and leadership opportunities (Kerrigan et al., 2019), offering a practical strategy to integrate perspectives from sex workers to customize C-PrEP + within the existing system, address local health needs, and enhance the likelihood of adoption and sustainability (Benoit et al., 2017). Focusing on the Exploration and Preparation phases of EPIS, this pre-implementation study was designed to inform pilot implementation by engaging clinic staff, care providers, and administrators.

This study reports on findings from the Exploration and Preparation phases of EPIS, including barriers and facilitators to sex worker care, PrEP usual care, Centering Healthcare, and Centering PrEP, from the perspectives of clinic staff and care providers, to inform the development of a successful implementation plan. Implementation and sustainability were not directly addressed, as this is a pre-implementation study. While these components are not detailed in this paper, the findings informed a pilot intervention blueprint to support future implementation and long-term sustainability (Singer et al., 2024). By engaging providers, administrators, and frontline staff early in the process, we aimed to lay the groundwork for successful adoption, iterative evaluation, and sustainment.

### Participants

The study was conducted from March 2022 to June 2022. We recruited clinic staff, providers, and administrators through clinic-based flyers, inter-office emails, and word of mouth. Potential participants were emailed or called by the study team, who assessed their eligibility and described the study. If eligible and interested, a remote study visit was scheduled. To be eligible, participants had to be (1) age 18 or older; (2) staff, care provider, or administrator directly involved with PrEP care at the Clinic; (3) speak and understand English; and (4) be willing and able to provide informed consent.

### Procedures

Using a semi-structured interview guide, developed in collaboration with community stakeholders for this study (see supplemental information), addressed familiarity with sex worker health, the standard of care PrEP protocol (PrEP usual care), Centering Healthcare, and feedback about C-PrEP + . Interviews informed a pilot implementation plan tailored to the needs of healthcare professionals who interface with patients who trade sex and seek PrEP for HIV prevention. Additionally, the guide contained questions and example probes but allowed the interviewer to add probes to explore topics naturally as they emerged. For instance, we asked, “What can be done to make C-PrEP + as accessible as possible for patients?”

After providing informed consent to participate in a Zoom-based interview, participants engaged in 45-min sessions conducted by the first author (RBS), a nurse practitioner, to share feedback about group PrEP care for sex workers. Rigorous measures ensured privacy and confidentiality, including secure links, encrypted communications, and explicit participant consent (Bhalerao et al., 2022; Mithani & Judge, 2024). Staff and clinicians were interviewed about barriers and facilitators to C-PrEP + implementation in the clinic setting, as well as organizational structure and usual challenges related to PrEP care. Questions such as “What is the most challenging part of your job?” highlighted challenges within the healthcare system and perceptions of how group care might improve workflow for care providers and staff. Final questions probed how participants would improve PrEP care to inform future PrEP interventions within this clinic. Interviews were recorded and transcribed. All interviews were conducted outside of work hours to mitigate any potential burden on the healthcare system. Additionally, given the clinic’s ongoing system-wide restructuring at the time of interviews, researchers made efforts to minimize disruption to daily operations. Participants received a $50 e-visa gift card for their time.

### Analysis

Data were analyzed using directed content analysis (Hsieh & Shannon, 2005). We utilized directed content analysis, guided by the EPIS framework, to understand provider, staff, and administrator perceptions of a group care intervention to support PrEP use among individuals engaged in sex work. Because EPIS focuses on identifying contextual factors, organizational readiness, and community-clinic alignment before implementation, this framework provides a structure to guide the content analysis, supporting a feasible, acceptable, and sustainable intervention blueprint (Moullin et al., 2019). Furthermore, because this study is grounded in community empowerment principles, it employs directed content analysis, focusing on centering the voices of clinic stakeholders before piloting the intervention (Wei & Johnson, 2019). Two individuals (RBS, CLP) with extensive qualitative expertise and a trained research assistant (SJ) coded the data. A codebook was developed through an iterative process that involved the interview guide (see supplement) and an initial review of transcripts (Braun & Clarke, 2006). The primary coder applied initial codes to a subset of transcripts, which all coders subsequently reviewed and refined based on emerging data patterns. The codebook underwent continual updates with defined terms and illustrative quotes. We utilized Dedoose, an adaptable online mixed-methods platform (Davey et al., 2010), and employed directed content analysis techniques (Hsieh & Shannon, 2005) to discern themes and sub-themes. A consensus was reached among all qualitative coders through discussion and iterative review processes. We shared the preliminary findings with the study team and the community advisory board, comprising current and former sex workers, healthcare providers, and researchers, for verification of major and minor themes, diverse and outlier perspectives, and validation of relevance. Saturation was determined by patterns in themes, consistent repetition, and limited emergence of new topics (Hsieh & Shannon, 2005).

## Results

### Participant Characteristics

We conducted 14 individual interviews with healthcare professionals, including administrators, registered nurses (RNs), primary care providers (PCPs), and PrEP navigators (see Table 1). These participants offered a range of perspectives, reflecting diverse roles, responsibilities, and viewpoints across the healthcare system.

We identified five themes related to perceived barriers and facilitators of caring for sex workers among healthcare professionals: Healthcare for sex workers, PrEP usual care, Centering Healthcare, Centering PrEP care (C-PrEP +) for sex workers, and access to usual PrEP or C-PrEP + care. These themes were selected based on their recurring presence during the coding of individual interviews.

### Healthcare for Sex Workers

In our formative work, this clinic was identified by sex workers as a trusted clinic in part because of its commitment to LGBTQIA+ sexual health and HIV prevention care (Singer et al., 2021). Because this study aims to facilitate culturally safe group PrEP care for those who trade sex, we asked all clinic staff and care provider participants (n = 14) about their experiences working with sex workers. When asked about caring for sex workers, participants discussed these patients’ increased HIV/STI vulnerability and HIV prevention. One RN highlighted HIV/STI acquisition risk factors, focusing on harm reduction.

I think mostly we look at risk factors. Who are you having sex with? How often are you having sex? Are you having sex with people you don’t know? Are you having transactional sex? If you’re saying you’re having sex with 40 people a month, then you’re considered high risk. We always want to recommend condom usage as much as possible, especially for transactional sex, because you don’t know… You don’t know anything about those people. There’s not even an assumed monogamy or anything like that—definitely condom usage. PrEP is definitely in the conversation for preventative health if they are HIV-negative.

(25-year-old, Multiracial, cisgender, questioning woman)

Additionally, PrEP professionals acknowledged the stigmatizing experience that frequently corresponds with disclosing sex work to care providers. Another RN, who works in the sexual health walk-in clinic, expressed his disappointment and surprise at how sex workers are perceived by his colleagues and other healthcare professionals.

There’s a lot of negative conception, which is very unfortunate because sex work is work. Sometimes, you do still see biases coming from providers, even here… There is a huge stigma surrounding sex workers across all levels of care. Techs, doctors, nurses. It’s actually kind of sad.

(26-year-old, white, cisgender, gay man)

### PrEP Usual Care

The theme of PrEP usual care describes all the normal activities associated with prescribing and managing care for patients taking PrEP. Codes in our data used to develop this theme include initial visits, laboratories, scheduling follow-up appointments, navigating insurance, and PrEP decisionmaking related to oral or injection-based PrEP, as well as improving onboarding and continuous procedures to foster PrEP care. An essential component of PrEP usual care, as described by providers and staff, was the importance of initial appointments for rapid PrEP start. Additionally, providers and staff expressed difficulties with patient retention after the onboarding component of PrEP usual care, as well as the structural care barriers that affect access to care, including transportation barriers and tight clinic scheduling. As one physician assistant described:

For PrEP… When I do the rapid start, I do all their screenings at that time for STIs, Hepatitis, their kidneys, liver, HIV testing, syphilis, all of that. And then I want them to come back in a month. That one-month appointment is super important because it picks up any remaining HIV that we couldn’t pick up on the testing we did that day of rapid start…I stress that, but for some people, it can be hard to get in.

(26-year-old, Asian, cisgender, heterosexual woman).

Despite the essential need for a one-month follow-up after PrEP initiation, staff and care providers report that patients new to PrEP had trouble scheduling return visits due to fewer prescribing provider openings. Staff and providers, seeking a magical solution, described how frustrating it is to have reduced provider availability and capacity. One RN, who consistently works in the sexual health walk-in clinic, expressed their vision of flexible scheduling that prioritizes patients’ day-to-day lives.

Magic wand’s perfect scenario: we would have just way more provider availability so that patients could just schedule those appointments. And then, because we had way more providers and way more availability, we would have a much wider grace period because I think it becomes difficult for patients to get with that 15-minute grace period. But because clinics are so swamped, you can’t operate without it, basically. And so, I think relaxing those late periods is really helpful for patients who are taking the bus or just have shit going on.

(33-year-old, white, non-binary and queer)

To improve access to usual PrEP care, participants identified that expanding provider capacity by having a full staff, including a PrEP navigation team, would increase organizational capacity to support the scheduling needs of patients prescribed PrEP.

### Perception of Centering Healthcare

Both healthcare providers and staff were asked about their knowledge of Centering, an evidence-based group healthcare model that benefits from community-empowered approaches. Afterward, all participants (n = 14) were informed about the Centering Healthcare structure and shown a visual to illustrate how individual health assessment, interactive education, and community building were integral to the success of this group care model. Only one participant currently provides Centering Care. All participants were told how Centering originated as a group prenatal care model but has since been successfully adapted to meet the needs of other populations, such as individuals with sickle cell disease and diabetes. Before addressing how we adapted Centering to meet the needs of sex workers on PrEP, we wanted to get feedback related to group care in general.

While several participants were familiar with Centering Healthcare, only one care provider had direct experience with Centering. A physician and Centering Pregnancy facilitator, she expressed why she believed outcomes for pregnant patients improve with Centering Healthcare.

It’s an opportunity for more education for the patients. It’s an opportunity for patients to learn from their peers. It’s an opportunity for us as facilitators to correct any myths that are out there. I think primarily, the good thing about Centering is the education and the discussion that ensues. What’s clear is that we need to educate our patients about their bodies and things that are going on, so that they can take an active role in prevention.

(56-year-old, Black, cisgender, heterosexual woman)

We inquired about factors that would encourage the adoption of group care. Staff and providers shared that the Centering model’s fundamental elements would offer patients a unique chance to connect through community building. Creating intentional opportunities for peer and expert support, fostering interactive learning, and facilitating social interaction would encourage community building. Additionally, participants highlighted the benefits of "Talking Openly" and the provision of a "Safe Space" as common advantages of group care. Providers and staff appreciated how the model aims to disrupt power dynamics inherent in current healthcare models. A community-engaged supervisor who oversees caseworkers articulated how needed community support would align with such a model.

I really like this already… A sense of community. I think that would really help patients. A lot of the times, I do feel like people feel alone … that they do feel isolated. It does help someone to understand that other people are going through the same thing as they are. Again, it’s just so different because in a group of people, they may feel more comfortable at times speaking up as opposed to being by themselves with a provider or a healthcare professional, where they may feel targeted, or judged, and this is their peers. I think that’s wonderful…It seems like it helps with empowering someone a little bit more…the idea of community that helps with accountability as well.

(43-year-old, Latinx, cisgender, heterosexual man)

### Perceptions of C-PrEP +

After explaining the Centering model, we delved into how we collaboratively adapted Centering to meet the PrEP needs of sex workers. We showed all participants a breakdown of the anticipated sessions, corresponding clinical and community-building activities, and the visit timeline to get their feedback on C-PrEP + . We asked all participants to share their insights about potential barriers and facilitators to C-PrEP + . While enthusiasm for C-PrEP + was evident, participants identified several barriers to its implementation. Foremost among these was the concern for "Privacy," as they expressed that some patients might hesitate to share sensitive information or disclose sex work in a group setting. One nurse practitioner said:

I think the biggest struggle would be maybe finding patients who are okay with people knowing their business. I think that’s where you would run into the most trouble, like finding some people who are open to saying, "Yes, I am a sex worker. And yes, I do this for money," in front of other people outside who they don’t perceive as their healthcare professionals or that they perceive as people on the streets or someone they may know. I think that may be the only issue you run into. There are some who are open to talking about it because they’re thinking, "If I’m here, you’re here too. We’re on the same playing field." But there are some who are a little more reserved and who may not be as open to discussing that.

(32-year-old, Black cisgender, gay man)

Additionally, despite the single-visit experience, which included laboratories, prescriptions, and a health assessment, some participants expressed apprehension regarding the time commitment associated with longer appointments. Such apprehension highlights how a two-hour all-inclusive visit may be perceived as too long despite being less than the actual care experience, which involves the clinic, laboratory draw, and scheduling wait times. One RN explained:

I think that for some people, PrEP is just very routine for them. It’s just an appointment that they have to keep and it’s not such a big part of their life and they want to keep it as small a part of their life as possible, so maybe… they would feel weird about having this much time dedicated to it.

(33-year-old, Asian, Cisgender, gay man)

However, despite the lengthier visit, perceptions of C-PrEP + were predominantly positive, focusing on community building and one-stop shopping, which would save both provider and patient time. Peer-to-peer involvement paves the way for accountability that would not be available in the individual 15-min visit model. One RN stated, “Treating that community, making friends, making people who hold you accountable, have you support, and then that repeatedly gets you to show up because it is a community model.” (26-year-old, white, cisgender, gay man)

Additionally, administrators and providers discussed the importance of in-group knowledge and how having such a community-empowered healthcare experience would enhance patient knowledge and self-care, as well as reduce the care provider’s burden in understanding the lived experiences of particular patients. One PrEP administrator expressed:

This is a space where everybody might have similar experiences. So instead of you asking a question about an STI, somebody else might ask it first, and then you can build on it… You’ll be in a community, you’ll be in a space where if you don’t know how to ask it, maybe you can start, and somebody else might jump in and say, "Is this what you’re referring to? Are you talking about a sore that you had, or are you talking about," I don’t know, "What lubricant to use that doesn’t tear up the latex? Is this an issue that you’re having? Because this is an issue that I had." So, they might feel a little more empowered to share information, as opposed to being by yourself in a doctor’s office who might not even know how to talk to you about these things.

(38-year-old, Latinx, cisgender, heterosexual woman)

One nurse practitioner acknowledged that she does not always have the answers when patients ask population-specific questions. Group care, she admitted, would offer patients the understanding needed to feel seen in a healthcare setting, even when the provider may lack cultural awareness.

So, I think it’d be great to hear other people’s concerns too. I’m not alone in thinking this way. Or how are other people protecting themselves in these types of situations? Especially with sex work, there could be safety concerns and other things, like what do I do in this situation? And I may not even know that answer. So, I have to lean to patients to educate me too. So, I think as a provider, it would be really valuable to be in this group setting and learn from the community because I don’t have all the answers. Definitely not. And sometimes, I don’t know, and I have to ask others. So, it’s kind of nice to combine everyone together and learn from each other.

(34-year-old, Asian, cisgender, heterosexual woman)

In addition to the community-building aspect, some participants appreciated the longer appointment times and one-stop-shopping elements of the model, which allowed for greater efficiency for both the clinic and the patient. For example, seeing eight to twelve patients at once for a more extended appointment period would save providers time and energy and enhance the patient care experience. When asked if instituting C-PrEP + would add to her workload, one staff member who handles scheduling, billing, and ordering laboratories expressed that C-PrEP + may reduce her workload burden.

I’m not sure that it would add (to the workload). If anything, it may streamline it…For instance, if we’re having Centering around PREP…it gives me an opportunity to even build a work queue so that I can look at all the patient’s billing, make sure it’s all correct, make sure it all goes out correctly at the same time, make sure all the labs are coded correctly. So, it may make my job easier as opposed to adding to it.

(51-year-old, multiracial, cisgender, heterosexual woman)

Additionally, a common barrier to patients getting on or continuing PrEP is provider availability, and the Centering model of care offers an alternative approach to managing scheduling appointments. After seeing a flowchart of the model and its associated activities, some staff and care providers expressed eagerness to offer C-PrEP + as an alternative to usual PrEP care. One RN said:

Oh, I think I would love it…I think it would be great for managing follow-up, managing long-term care, getting people to come into clinic to be seen. And what’s nice about it is, like I said, monthly complaints we have, one of the barriers we have is sometimes provider availability

(26-year-old, white, cisgender, gay man).

### Improving Accessibility and Interest in C-PrEP +

At the end of the interview, we asked participants about recommendations for improving the accessibility of C-PrEP + . Participants offered recommendations to enhance the accessibility of and interest in the Centering model, with food emerging as the most popular incentive to encourage patient retention. Others suggested incentives included vouchers, transportation, and marketing of PrEP availability. An RN who works with drop-in patients discussed enticements for patient engagement.

At the end of the day, if you don’t have coverage, even with 340B pricing, it can still be about $20 a bottle. Maybe someone doesn’t have $20 a bottle to pay for certain services or things like that. I would say definitely voucher programs to make sure that once we do all of this, we’re still able to get people on PrEP if money is a financial barrier. Food. Food at these little meetings. Everyone loves food. Entice them in with food.

(26-year-old, white, cisgender, gay man).

Overall, participants expressed optimism about the potential of the one-stop shopping explicit to C-PrEP + , recognizing its ability to alleviate the burdens associated with fragmented healthcare services and offering a holistic and streamlined approach to care delivery. One RN shared her favorable views of C-PrEP +:

I do like it because I think it’s overwhelming for patients to keep coming back and they have to get labs drawn and they have to see a doctor. Sometimes these are separate things … I think it’s a nice that it’s a one-stop shop model: talk about it, meet other people, get your labs drawn, do a urine dip

(25-year-old, Multiracial, cisgender, questioning woman).

## Discussion

Our study provides important insights into the barriers and facilitators for integrating group PrEP care for sex workers within existing clinical structures. Currently, models of PrEP care used by sex workers are often based in multidisciplinary community healthcare settings, such as Clinics. Therefore, it is essential to understand healthcare professionals’ perceptions of potential obstacles and enablers for implementing a Centering model for PrEP care (C-PrEP +) in these community settings. This understanding will help ensure that implementation is guided by key stakeholders, leading to successful and sustainable adoption. Healthcare professionals’ perceptions will shape future efforts to improve access to comprehensive PrEP care for sex workers. They needed access to comprehensive PrEP care for sex workers.

PrEP care requires engagement with multiple healthcare professionals, such as administration, insurance, phlebotomists, and prescribers (Pathela et al., 2020). Individuals within the Clinic who represent these various roles bring essential insights into the barriers and facilitators of PrEP care. With representatives from multiple healthcare roles, including administrators, registered nurses, primary care providers, and PrEP navigators, the findings encapsulate a multifaceted understanding of the complexities inherent in providing care for individuals at increased vulnerability to HIV, particularly those engaged in sex work (Calabrese, 2020; Cooney et al., 2023; Ma et al., 2017).

In our study, and consistent with other research, we noted that fostering trust and implementing a harm reductioncentered standard of care was pivotal in providing practical support for sex workers (Benoit et al., 2019; Singer et al., 2021). This approach prioritizes the well-being of all individuals involved rather than focusing solely on risk assessment, thereby mitigating the potential for stigmatization, particularly within healthcare provider interactions (Johnson et al., 2023).

Our findings regarding PrEP usual care revealed facilitators and barriers in preparation for service implementation. The presence of a care coordinator or PrEP navigator emerged as a facilitator, fostering follow-up and enhancing patient engagement (Pathela et al., 2020). However, barriers such as limited availability of prescribing providers underscore the importance of thorough preparation to address logistical challenges and optimize implementation. This underscores the significance of the Preparation phase within the EPIS framework, where strategies are developed to mitigate potential service delivery challenges and maximize program effectiveness (Moullin et al., 2019).

The concept of centering care received mixed reactions from participants. While community building and peer relationships were identified as potential facilitators, concerns regarding privacy, time commitments, and logistical barriers were seen as barriers. These findings highlight the importance of tailoring care models to specific group preferences and needs, ensuring that patient-centered approaches remain at the forefront of group care delivery (Catalyst, 2017; Larmarange et al., 2018).

Transitioning to the C-PrEP + model was met with enthusiasm among participants, who recognized its potential to facilitate community empowerment and streamline care delivery (Benoit et al., 2017; Kerrigan et al., 2015). Facilitators such as community building and peer relationships were identified, along with creating a "one-stop shop" for patients and providers (George et al., 2020). Providers appreciated the patient-centered nature of C-PrEP + , valuing the idea of patients as experts in their care (Abubakari et al., 2021; Ayala et al., 2021; Benoit et al., 2017a, 2017b).

However, several barriers were noted during interviews. Concerns regarding confidentiality and disclosure within group settings emerged as a barrier, highlighting the need for privacy safeguards (Friedman, 2015). Additionally, the amount of time spent on PrEP care was cited as a barrier, with the two-hour duration considered too long for some individuals.

Addressing these barriers will be crucial in ensuring the successful implementation of the C-PrEP + model. Strategies to enhance confidentiality, such as implementing strict privacy protocols, should be prioritized. Likewise, efforts to streamline care processes and reduce time commitments, perhaps by offering flexible scheduling options or shorter session durations, are essential. By proactively addressing these barriers while leveraging facilitators such as community engagement and patient empowerment, healthcare providers can maximize the effectiveness of the C-PrEP + model and enhance its impact on HIV prevention efforts (Ma et al., 2017; Neely et al., 2021).

Our study identified several strategies to enhance accessibility and engagement within the C-PrEP + model. Participants highlighted the value of incentives, including food vouchers, transportation assistance, and flexible scheduling options. These recommendations underscore the importance of proactive measures and community resources in overcoming barriers to care, ultimately facilitating greater engagement and retention in PrEP services (Roy Paladhi et al., 2022).

Our findings shed light on the complex interplay between structural barriers, patient preferences, and care delivery models in supporting sex workers’ access to HIV prevention services (Cooney et al., 2023; Kerrigan et al., 2015; McCall, 2005). Innovative approaches such as C-PrEP + must address the logistical and privacy concerns associated with group care. In alignment with best practices in implementation science, our study aims to address these barriers proactively by incorporating solutions within the implementation plan. By leveraging community resources, C-PrEP + may offer an empowering alternative to traditional PrEP care, cultivating a supportive environment for sex workers that encourages community building, health promotion, and PrEP retention (Blachman-Demner et al., 2017; Larmarange et al., 2018; Rousseau et al., 2021).

### Study Limitations and Implications for Future Research

While our study provides valuable insights into healthcare providers’ perspectives on Group PrEP care for sex, several study limitations exist. This study was limited in scope as it focused specifically on healthcare professionals’ perspectives and experiences with PrEP care for sex workers within the context of a single clinic. As a result, our findings may not fully capture the perspectives of other key stakeholders, such as sex workers themselves or administrators from different healthcare settings. The sample size of 14 participants, although diverse in terms of professional roles and demographic characteristics, may not fully represent the breadth of perspectives within the healthcare provider community as a whole. Care professionals recruited from this specific clinic may be familiar with sex worker rights or supportive of innovative care models such as Centering PrEP care (C-PrEP +), introducing potential bias. Furthermore, the willingness to participate in the study may indicate a higher level of interest or engagement in PrEP care for sex workers, potentially skewing interview perspectives (Pleuhs et al., 2020). The data collected in this study primarily relied on self-reported information obtained during individual interviews. As such, social desirability bias or inaccuracies in recall may have influenced the data obtained (Bergen & Labonté, 2020).

While valuable for exploring nuanced perspectives and experiences, the qualitative nature of our study limits the broader applicability (Degtiar & Rose, 2023). Further research employing quantitative methods and larger sample sizes could provide additional insights and confirm the validity of our findings (Hennink & Kaiser, 2022; Lakens, 2022). Despite these limitations, our study contributes to the growing body of literature on PrEP care for sex workers. It offers practical implications for improving the design and delivery of preventive services. Our findings provide valuable insights for healthcare providers, administrators, and policymakers who seek to foster health promotion through policies informed by sex workers and to expand access to culturally safe preventative healthcare for sex workers and other vulnerable and stigmatized populations.

## Conclusion

Findings from this study informed the development of a C-PrEP + pilot implementation plan. The implementation of C-PrEP + holds promise for impacting sex workers across interpersonal, intrapersonal, community, and institutional levels. By integrating comprehensive support, C-PrEP + has the potential to empower sex workers, reduce HIV/STI transmission, and enhance overall health outcomes. Positive patient experiences, efficient use of provider time, and improved HIV outcomes associated with Centering models further highlight the benefits of adopting such approaches. By directly engaging with sex workers and community healthcare providers, C-PrEP + addresses structural barriers such as access to care, stigma, and healthcare discrimination. Moreover, it caters to the diverse needs of sex workers regarding PrEP adherence, sexual health, disease prevention, and contraceptive needs through community-engaged support.

In essence, the findings of this study underscore the significance of centering the needs and perspectives of individuals at heightened vulnerability to HIV in PrEP service design and delivery. Embracing innovative models like C-PrEP + and addressing systemic barriers is pivotal for advancing health equity and promoting the well-being of diverse communities. Continued collaboration among healthcare providers, community stakeholders, and policymakers will be instrumental in realizing the full potential of C-PrEP + and other patient-centered, community-empowered approaches to HIV prevention for sex workers.

## Figures and Tables

**Table 1:** Participant demographics

	n	%
*Position*		
Administrator	4	29%
Registered nurse (RN)	4	29%
Primary care provider (PCP)	4	29%
PrEP navigators	2	14%
*Gender identity*		
Cisgender man	5	36%
Cisgender woman	5	36%
Genderqueer/gender fluid	3	21%
Transgender	1	7%
*Race*		
White	4	29%
Latinx	3	21%
Multiracial	3	21%
Black/African-American	2	14%
Asian	2	14%
*Education*		
Some college	1	7%
Associate’s degree	2	14%
Bachelor’s degree	5	36%
Graduate degree	6	43%

## Data Availability

The datasets used and/or analyzed during the current study are available from the corresponding author on reasonable request.
